# Serum thyroglobulin is associated with orbitopathy in Graves’ disease

**DOI:** 10.1007/s40618-021-01505-8

**Published:** 2021-01-29

**Authors:** S. Khamisi, M. Lundqvist, P. Emadi, K. Almby, Ö. Ljunggren, F. A. Karlsson

**Affiliations:** 1 Department of Endocrinology and Diabetes, Uppsala University Hospital, 751 85 Uppsala, Sweden; 2 Department of Medical Sciences, Uppsala University, Uppsala, Sweden; 3 Department of Ophthalmology, Uppsala University Hospital, Uppsala, Sweden

**Keywords:** Thyroglobulin, Graves’ disease, Graves’ orbitopathy, Graves’ ophthalmopathy

## Abstract

**Purpose:**

Serum thyroglobulin levels are often elevated in Graves’ disease (GD) and in most cases decrease during treatment. Its relation to Graves’ orbitopathy (GO) has not been clarified. Previously, a risk of GO has been linked to smoking, TSH receptor stimulation, high TSH-receptor antibodies (TRAb), low thyroid peroxidase and thyroglobulin antibodies (TPOAb, TgAb).

**Methods:**

We examined Tg levels in 30 consecutive patients with GD were given drug therapy (methimazole + thyroxine) for up to 24 months. GO was identified by clinical signs and symptoms. 17 patients had GO, 11 of whom had it at diagnosis while 6 developed GO during treatment. During the study, 5 subjects were referred to radioiodine treatment, 3 to surgery. The remaining 22 subjects (GO *n* = 12, non-GO *n* = 10) completed the drug regimen.

**Results:**

At diagnosis, Tg levels in GO patients (*n* = 11) were higher (84, 30–555 µg/L, median, range) than in non-GO patients (*n* = 19) (38, 3.5–287 µg/L),* p* = 0.042. Adding the 6 subjects who developed eye symptoms during treatment to the GO group (*n* = 17), yielded *p* = 0.001 vs. non-GO (*n* = 13). TRAb tended to be higher, while TPOAb and TgAb tended to be lower in the GO group. For the 22 patients who completed the drug regimen, Tg levels were higher in GO (*n* = 12) vs. non-GO (*n* = 10), *p* = 0.004, whereas TRAb levels did not differ.

**Conclusion:**

The data may suggest that evaluation of thyroglobulin levels in GD could contribute to identify patients at increased risk of developing GO. Possibly, thyroidal release of Tg in GD reflects a disturbance that also impacts retroorbital tissues.

## Introduction

Thyroglobulin (Tg) is produced by the follicular cells of the thyroid gland and secreted into the apical lumen of the follicles where it is concentrated and used as a substrate for the synthesis of thyroid hormone [1]. Small amounts of Tg are released from the thyroid under physiological as well as pathological conditions. For example, Izumi and Larsen [2] found elevated serum Tg levels in untreated Graves’ disease (GD) patients, 132 ± 124 μg/L (mean ± SD) as opposed to 11 ± 6.4 ug/L in healthy subjects and observed elevations in serum Tg up to 7000 μg/L within 24–48 h after subtotal thyroidectomy or 131I treatment of GD. Pacini et al. [3] reported serum Tg in normal individuals to be 9.5 ± 0.9 μg/L (mean ± SE), in untreated and treated subjects with diffuse toxic goiter 424 ± 101 and 328 ± 222 ug/L, respectively, in nontoxic goiter 61.4 ± 15 μg/L, in untreated differentiated thyroid carcinoma 89.5 ± 19 μg/L and in subacute thyroiditis 138 ± 67 ug/L. Madeddu et al. [4] studied 12 patients with subacute thyroiditis over 3–4 months, ten of whom demonstrated similar patterns with initially elevated Tg values 300 ± 204 ug/L (mean ± SD, a decline to 43 ± 21 ug/L within 20 days of steroid therapy, a further decline to normal values for 20 days and then a rise above normal for approximately 40 days, eventually returning to the normal range. Interestingly, Druetta et al. [5] in a skillful study showed that Tg molecules released in subacute thyroiditis are iodinated, i.e. contain thyroid hormones and likely reflect a destruction of thyroid follicles. In contrast, non-iodinated Tg molecules were found in the circulation of seven out of eight untreated GD patients. The mechanism behind the release of non-iodinated Tg has not been clarified.

Increased serum levels of Tg that tend to fall during anti-thyroid drug (ATD) treatment have been observed in patients with Graves’ disease with no simple relationship to the extent of thyrotoxicosis. Uller et al. [6] studied 34 GD cases. Patients whose GD relapsed had higher Tg levels both before and after ATD, while the levels were lower and dropped in patients that went into remission. Aizawa et al. [7] examined 113 patients with hyperthyroidism who received ATD for 10 months and subsequently a T3 suppression test. Cases with a positive suppression test had significantly lower Tg at the time of the test. Gonzàlez -Jimènz et al. [8] studied 51 patients with GD and noted that in relapses vs early remissions the changes in antithyroid antibodies and serum Tg after 6 months of methimazole therapy were different. At the termination of ATD, serum levels of Tg > 75 ug/L and TRAb > 30 U/l (ref < 15 U/L) were considered markers predicting GD recurrence. Gong ST et al. [9] studied changes in Tg during the course of ATD in 65 patients and found that the Tg levels were significantly higher in the relapse group than in the remission group both before and after ATD. Werner et al. [10] in a cohort of 42 patients with GD reported a non-significant difference in Tg concentrations between patients who were in remission or relapse, 132 ± 242 (mean ± SD) vs. 214 ± 235 ug/L, respectively.

Graves' orbitopathy is clinically relevant in approximately 50% of patients with GD, with severe forms affecting 3–5% of patients [11]. In patients without clinically apparent ophthalmopathy, radiological signs of muscle enlargement have been found in 40% [12]. Several studies have been conducted to predict the risk of recurrence of GD. Smoking [13] and TSH receptor stimulation [14–16] are known risk factors for GO. Furthermore, high TRAb levels have been associated with an increased risk of GO [17] as have low levels of TPOAb [18–22] and lower TgAb [19] [22]. In one study, higher TgAb [20] levels were linked to risk of GO. For evaluation of Tg in relation to GO, Lahooti et al. examined a group of 54 patients with GD, out of whom 23 patients had GO. 52% of patients with GO had elevated levels of Tg compared to 26% in the group with no GO [23].

Early diagnosis and treatment of GO may in most cases protect against the development of complications and sequelae from severe GO. At present, Tg is not used as a marker to predict either recurrence risk or risk of GO. In this paper, we prospectively studied patients with newly diagnosed GD to investigate differences in Tg levels at diagnosis and during treatment depending on GO status.

## Materials and methods

### Study subjects

Thirty patients with de novo Graves’ disease, diagnosed with decreased levels of TSH and positive TRAbs, were recruited at the Uppsala University Hospital during February through November 2017. Their median age was 55 years (range 35–72 years), 2 were smokers, 29 were women. Four patients were on betablockers, three on selective serotonin reuptake inhibitors and one on thyroxine. Ultrasound was performed in one case with a palpable thyroid nodule and showed benign findings*.* One patient had a TRAb below the reference range (1.7 IE/L, reference < 1.75). This patient otherwise had symptoms and laboratory findings typical for GD, which was in line with a homogeneous uptake at scintigraphy.

The study consisted of 6 visits. During the first visit (v1) all patients underwent an examination including the recording of demographic characteristics, medical history, family history and concomitant medication. Blood sampling to measure TSH, fT4, fT3, TRAb, TPOAb, TgAb and thyroglobulin was performed at baseline (v1), and 6 weeks (v2), 12 weeks (v3), 6 months (v4), 12 months (v5) and 24 months (v6) after treatment start. In all 30 subjects, anti-thyroid drugs, methimazole or propylthiouracil, were initiated in conjunction with visit 1 and given in a block-replace regimen, beginning with 10–20 mg methimazole doses or 150–300 mg propylthiouracil. When thyroid hormone levels fell into the normal range, thyroxine 50–100 μg daily was added and TSH levels kept in the low normal range. Throughout, care was taken to avoid treatment-induced episodes of hypothyroidism.

The medical regimen was altered in eight subjects, who were referred to radioiodine treatment (*n* = 5) or surgery (*n* = 3). Four patients received radioiodine (RAI) because of persistently elevated TRAb after 10–15 months ATD treatment. One patient received RAI after 5 months at the request of the patient. Post-RAI, one patient experienced mild GO, not requiring steroid medication. Three patients underwent total thyroidectomy. For two patients, this was due to neutropenia, which developed directly after start of methimazole in one case and after 10 months on methimazole in the other. One female patient was operated after 5 months because of pregnancy desire. Out of the remaining 22 subjects, 21 patients received ATD for 18–24 months until negative TRAb. One non-GO patient had a spontaneous recovery before treatment initiation.

### Evaluation and classification of GO

All patients received clear information about GD and they were examined at each contact by a doctor and a nurse with experience in managing GD regarding the presence of eye symptoms and signs and were repeatedly told to contact the clinic in the case of eye symptoms. GO was defined as eye symptoms and signs related to GD and classified as ‘mild’ GO with symptoms such as gritty sensation and tearing due to dry eyes, caruncle swelling and/or redness, upper eyelid retraction, or as ‘moderate to severe’ in the instance of redness and/or swelling of the eyelids, chemosis, pressure or pain in the eyes, exophthalmos, diplopia or signs of optic nerve compression. The eye signs and symptoms were detailed in the medical records by the attending endocrinologist as well as by the research nurse at every visit. To assess the severity of GO we used the EUGOGO classification [24]. Three patients with moderate to severe GO were referred to an ophthalmologist, who also examined and advised on 6 mild cases. The mild cases had mostly mild tissue involvement and corneal exposure responsive to lubricants. In the moderate to severe GO group, one patient presented signs and symptoms already at visit 1. This patient had a unilateral exophthalmos with eyelid retraction, redness and diplopia. One patient had increasing eyelid swelling at visit 2 and one patient developed a unilateral exophthalmos at visit 5. Subjects were divided into an “all GO” group (*n* = 17), including patients who at diagnosis presented (*n* = 11) or during treatment (n = 6; v2, v3, v3, v4, v5 and v5) developed eye signs and symptoms typical of GO and a “non-GO” group of patients without any signs or symptoms of eye disease (*n* = 13) throughout the study period. Out of the 22 subjects who were not referred for surgery or RAI during the study period, 12 were classified as GO (at diagnosis or during treatment) and ten as non-GO.

## Assays

Plasma TSH (reference interval 0.4–4.0 mIU/L, free T4 (reference interval 12–22 pmol/L, free T3 (reference interval 3.1–6.8 pmol/L, Tg (reference interval 3.5–77 µg/L), TRAb (reference < 1.75 IE/L), TPOAb (reference < 34 KIE/L), TgAb (reference < 115kIE/L) were measured with methods in routine use at the Department of Clinical Chemistry of the Uppsala University Hospital. The method used for measuring Tg in serum was the immunometric Elecsys Tg II assay by Roche on the Cobas instrument (Roche Cobas8000, e602). Tg concentrations in subjects without hyperthyroidism fall within the reference range 3.7–77 ug/L, based on measurements of 478 healthy Caucasian people (224 women and 254 men).

### Statistical analysis

All statistical analyses were performed using SPSS version 25. Graphs were constructed using Graph Pad Prism version 8.3.0. Data are presented as median (range) unless otherwise indicated. *P* values < 0.05 were considered significant. Since the main endpoint of the study was Tg levels at baseline and during follow-up, and the other thyroidal biomarkers were only analyzed to put possible Tg findings into perspective, no correction for multiple testing was undertaken*. *Baseline data at the first visit were compared between GO and non-GO groups using Mann–Whitney *U* Tests. For all subjects who did not undergo surgery or RAI (*n* = 22), the area under the curves of Tg and TRAb for the 24-month follow-up period were calculated with the trapezoid method and compared between the groups GO (*n* = 12) and non-GO (*n* = 10) using Mann–Whitney *U* tests. Missing values (1 for Tg, 11 for TRAb) were interpolated or extrapolated based on linear regression of adjacent values.

## Results

**Fig. 1:**
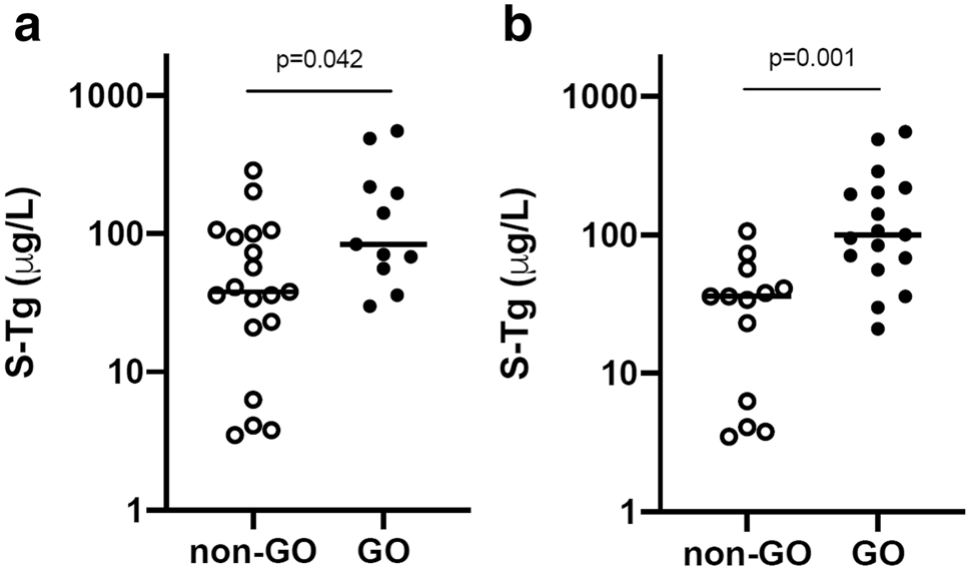
At baseline, Tg levels in GO patients (*n* = 11) were higher than in non-GO (*n* = 19) (**a**), *p* = 0.042. Adding the 6 subjects who developed eye signs during treatment into GO (*n* = 17) vs. non-GO (*n* = 13) (**b**), yielded *p* = 0.001

Thyroglobulin Tg levels differed significantly between subjects who presented with GO at the baseline visit (*n* = 11) and those who did not (*n* = 19) (Fig. 1a), while there were no differences in other thyroidal biomarkers (Table 1). Adding the 6 subjects who developed eye signs during treatment to create an “all GO” group (*n* = 17) and comparing them to non-GO (*n* = 13) enhanced the group difference for Tg (Fig. 1b). In addition, fT3 and fT4 were higher, TPOAb was lower and there were tendencies towards higher TRAb and lower TgAb in the all GO group vs. the non-GO group (Table 2). In the three patients who underwent total thyroidectomy, Tg dropped as expected post-surgery to very low levels, between 0.04 and 0.18 ug/L at visit 6. We found no significant correlations between Tg levels and TRAb, fT3, or fT4.

**Table 1:** Thyroid hormone, thyroglobulin and autoantibody levels (median and range) of 30 patients with de novo GD divided into GO and non-GO groups on the basis of GO present at diagnosis

	TSH	F-T4	F-T3	Tg	TRAb	TPO-Ab	Tg-Ab
All GD (*n* = 30)	0.005 (0.005–0.040)	41.0 (17.6–100.0)	15.6 (5.6–34.0)	62.5 (3.5–555)	5 (1.7–78)	28 (1.88–600)	14.5 (10–1354)
GO (*n* = 11)	0.005 (0.005)	48 (25–100)	18.5 (9–34)	84 (30–555)	9 (2.7–78)	22 (1.88–216)	11 (10–1354)
Non-GO (*n* = 19)	0.005 (0.005–0.040)	39 (17.6–85)	13.7 (5.6–30)	38 (3.5–287)	4.9 (1.7–29)	57 (7.6–600)	40 (10–1281)
*p*	0.350	0.232	0.158	0.042	0.216	0.372	0.445

Statistical differences between the two groups are given in the bottom line

**Table 2:** Thyroid hormone, thyroglobulin and autoantibody levels (median and range) at diagnosis of 30 patients with de novo GD divided into an all GO group, including 6 patients who developed GO after visit 1, and a non-GO group

	TSH	F-T4	F-T3	Tg	TRAb	TPO-Ab	Tg-Ab
All GD (*n* = 30)	0.005 (0.005–0.040)	41.0 (17.6–100.0)	15.6 (5.6–34.0)	62.5 (3.5–555)	5 (1.7–78)	28 (1.88–600)	14.5 (10–1354)
GO (*n* = 17)	0.005 (0.005)	48 (25–100)	18.3 (9–34)	100 (21–555)	6.3 (2.7–78)	12.1 (1.88–285)	10 (10–1354)
Non-GO (*n* = 13)	0.005 (0.005–0.040)	35 (17.6–60)	12.4 (5.6–28)	36 (3.5–106)	4.9 (1.7–29)	98 (8.2–600)	154 (10–637)
*p*	0.157	0.007	0.025	0.001	0.053	0.048	0.157

Statistical differences between the two groups are given in the bottom line

Twenty-two subjects (GO *n* = 12, non-GO *n* = 10) completed the ATD regimen. Table 3 displays hormone, Tg and thyroid autoantibody levels determined on 6 occasions during up to 98 weeks. As can be seen, hormone and autoantibody levels were numerically similar and changed similarly in both groups. The AUC of Tg was significantly higher in group GO *vs*. non-GO [2049 (144–5285) vs. 583 (29–1936) µg/L × months, *p* = 0.004], whereas the AUC of TRAb did not differ between the groups [41.8 (14.6–120.6) *vs*. 36.0 (10.5–171.6) U/L × months, *p* = 0.674].

**Table 3:** Longitudinal thyroid hormone, thyroglobulin and autoantibody levels determined in connection with 6 visits from 0 to 24 months in 22 patients with de novo Graves’ disease

Visit	GO	TSH	F-T4	F-T3	Tg	TRAb	TPO-ab	Tg-ab
0	GO	0.005 (0.005)	47.5 (25–85)	17.7 (9–34)	95.5 (21–287)	5.7 (2.7–20)	16.8 (7.6–285)	10 (10–1354)
	non GO	0.005 (0.005–0.04)	34 (17.6–60)	12.3 (5.6–28)	29.5 (3.5–73)	4.55 (1.7–29)	132 (12.3–600)	97 (10–413)
6 w	GO	0.005 (0.005–0.007)	2 (14.2–36)	6.5 (4.5–8.1)	104.5 (13–240)	7.25 (2.1–10)	15.6 (9.3–246)	10 (10–1314)
	non GO	0.005 (0.005–0.9)	21 (13.9–26)	5.2 (3–7.8)	19.5 (2.5–48)	5.05 (1.2–28)	115 (9–600)	86 (10–396)
12 w	GO	0.005 (0.005–5.68)	22 (11.7–33)	4.95 (3.4–12.6)	100.5 (4.2–274)	3.7 (1–7.8)	12.5 (7.5–158)	10 (10–1185)
	non GO	0.019 (0.005–3.56)	17.9 (12.5–29)	4.7 (3.4–6.4)	14 (1.8–50)	3.3 (0.7–19)	84.5 (11.4–600)	59 (10–375)
6 m	GO	0.202 (0.005–10.4)	17.2 (12.2–31)	4.1 (3.6–5.6)	104.5 (8.7–531)	1.4 (0.3–7.1)	13.1 (7.4–87)	10 (10–1128)
	non GO	0.86 (0.05–4.2)	17.35 (11–22)	3.85 (3.2–4.8)	18 (0.67–147)	1.5 (0.3–7.8)	62.5 (11.6–258)	40.5 (10–290)
12 m	GO	0.755 (0.04–4.67)	17 (12.8–25)	4.2 (3.4–4.6)	83 (3.5–186)	0.35 (0.3–3.8)	11.9 (9–103)	10 (10–1129)
	non GO	1.41 (0.3–4.4)	18.3 (14.7–24)	4.2 (3.8–6.1)	28 (0.9–79)	0.3 (0.3–2)	39.5 (10.8–159)	22.5 (10–252)
24 m	GO	2 (0.07–4.47)	16 (10–19.7)	4.6 (3.7–5.1)	32 (4.7–167)	0.3 (0.3–1.8)	14.1 (5–93)	10 (10–1091)
	non GO	1.79 (0.2–2.6)	15.6 (12.8–25)	4.6 (3.4–6.9)	17.5 (1.3–56)	0.3 (0.3–3)	45 (12.3–218)	31.5 (10–325)

The data (median and range) are presented in GO (*n* = 10) and non-GO (*n* = 12) groups. RAI and thyroidectomy were excluded

Fourteen patients presented with or developed mild eye signs/symptoms and three patients had moderate to severe GO. The three patients with moderate to severe orbitopathy received steroids, tapered over time according to clinical response. Two were treated with oral steroids, one after 11 months (i.e. one month prior to v5) received 30 mg prednisolone, tapered over 6 weeks (serum Tg at v1, v2, v3, v4, v5, v6 was 107, 144, 223, 279, 26, 71 μg/L, respectively). The other received 20 mg prednisolone tapered over 1 month (v2 to v3, serum Tg at v1, v2, v3, v4, v5, v6 was 21, 13, 4.2, 8.7, 3.5, 4.7 μg/L, respectively). One patient was given intravenous corticosteroids between v3 and v4 (500 mg and 250 mg methylprednisolone once a week for 6 plus 6 weeks) because of persistent GO despite prior oral steroids between v1 and v3, 20 mg prednisolone tapered to 7.5 mg over 3 months, compliance not documented (serum Tg at v1, v2, v3, v4, v5, v6, was 30, 51, 64, 25, 27, 100 ug/L, respectively).

## Discussion

In this study, we found that Tg levels were significantly higher both before and during treatment with ATD in patients with GO as compared to patients who did not develop GO. The difference in Tg levels was more marked than that of other thyroidal biomarkers. While TRAb levels tended to be higher at baseline, they normalized similarly in both groups and did not differ during treatment. Thus, it is unlikely that the observed difference in Tg levels was mediated by higher disease activity, as reflected by TRAb levels. We have no experience with the new assay of stimulating thyrotropin-receptor antibodies [27].

Most studies performed to evaluate the role of Tg in the assessment of disease activity in GD have shown that Tg may have a role in predicting the risk of recurrence [6–9]. We are aware of only one study that has investigated serum Tg in relation to GO [23]. Serum Tg levels were measured in 54 single samples from patients with GD. Increased levels were determined in 56% of sera from GO patients vs. 28% in samples from patients without GO. The same study also referred to the so-called Kriss hypothesis from the early 1970s [25] which stipulates that the reason for the development of GO is due to an accumulation of Tg in the orbital tissues, possibly through the lymph flow, leading to an autoimmune reaction against Tg bound to the extraocular muscles. However, later studies have suggested other pathological mechanisms to better explain the development of GO, making the Kriss theory controversial [26].

According to previous observations, thyroglobulin is elevated in thyrotoxicosis [2, 3, 6]. However, in our cohort, 11 of 30 patients with GD had a normal s-Tg at inclusion. Most patients displayed declining Tg levels months after discontinuing ATD (data not shown), which may allow Tg to be used as a marker for assessing disease activity even at normalized thyroid hormone levels and TRAb. To examine this aspect, larger studies with longer follow-up are needed.

**Fig. 2:**
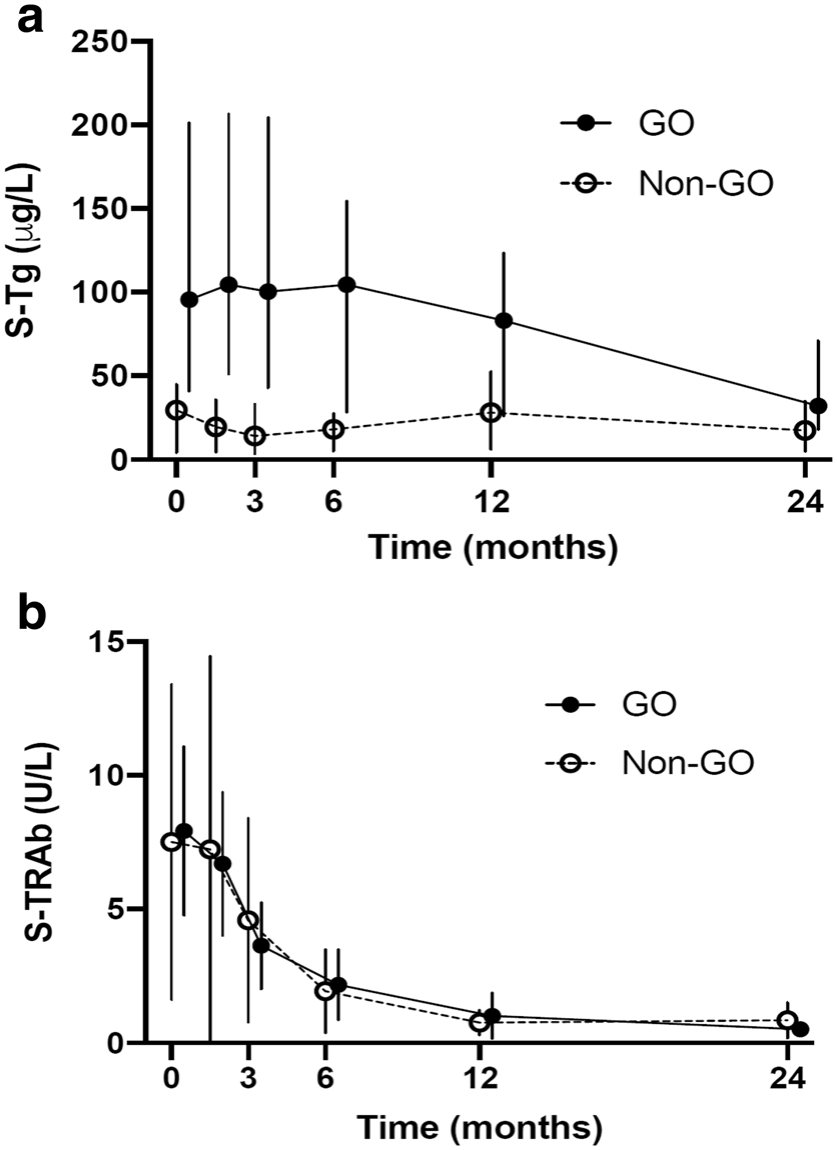
Tg and TRAb levels in all visits for the ATD group (*n* = 22) divided into GO (*n* = 12) and non-GO (*n* = 10), showing a protracted course for Tg (**a**) in GO vs non-GO group, whereas TRAb levels (**b**) were similarly reduced and normalized in both groups. Data presented as medians and interquartile ranges

In normal conditions, only small amounts of Tg leaks into the circulation, but Tg rises in some thyroid diseases such as GD. Tg might be released by a destruction of the follicular epithelium secondary to an inflammatory process. However, in the case of GD, Tg seems to be non-iodinated, in contrast to subacute thyroiditis which is characterized by cell destruction and causes release and elevation of iodinated Tg [5]. Possibly, newly formed non-iodinated Tg is secreted from transport vesicles in thyrocytes into the extrafollicular space by a mechanism that is controlled by TSH or TRAb [28]. In healthy individuals with an intact thyroid gland, rhTSH stimulation increased the median of serum TSH to more than 200 mU/L after 4 h and the circulating Tg increased 13- and threefold after 48 h and 28 days, respectively [29]. In the present study, Tg was still elevated despite a normalization of TRAb during the course of the disease (cf. Fig. 2b), suggesting mechanisms other than TSH-receptor stimulation behind the Tg elevation in GD. Another possibility which has been discussed is that proteins passes by a modification of the epithelium through the follicular cell wall via an intercellular route [30]. In addition, Tg may move from the follicular lumen through pinocytosis [31].

Steroids are still considered first-line treatment for active GO. The effect of steroids on GO involves inhibition of inflammation and a reduction in synthesis and secretion of glycosaminoglycans by orbital fibroblasts [32]. Three patients received steroid treatment, after which Tg levels decreased. In one of these cases, the oral treatment appeared insufficient, Tg levels decreased only following the administration of iv steroids. The majority of the patients in the all GO group (14/17) had merely mild symptoms and did not receive steroids. The clinical phenotype of GO nowadays is milder than in the past [33], likely due to reduced smoking habits and improved management of GD.

The small sample size of the present study should be considered when interpreting the findings. There is a need for confirmatory studies in larger cohorts, which should be powered for more advanced statistical methods such as regression analyses to investigate the independence of the association between Tg levels and GO status when adjusting for other thyroidal biomarkers. A larger cohort might also enable the development of a clinical scoring system to assess the risk of GO for the individual patient. The strength of the study is that it is prospective and that the included patients underwent continuous follow-up by both endocrinologist and nurse with experience of GD. Furthermore, ophthalmologists were consulted for assessment, characterization and follow-up for 9 of 19 patients with GO. All patients with newly diagnosed GD and were referred to our department during the period February-November 2017 were asked for participation in this study and all have agreed and are included in the study except 1 patient. Thus, there has not been any selection of patients to this study.

In conclusion, the data indicate that evaluation of thyroglobulin levels at diagnosis of GD could help identify patients at increased risk of developing GO and thereby impact the choice of therapy. Possibly, the thyroidal release of Tg in GD reflects a disturbance that also influences the retroorbital tissue in Graves’ orbitopathy. Further studies with larger cohorts are warranted.
